# Noncalcified Gallstone Ileus in Computed Tomography (CT) Abdomen and Pelvis With Contrast

**DOI:** 10.7759/cureus.70524

**Published:** 2024-09-30

**Authors:** Haseeb Qureshi, Mona Sabala

**Affiliations:** 1 Radiology, Surrey and Sussex Healthcare NHS Trust, East Surrey Hospital, Redhill, GBR

**Keywords:** ct, gallstone, ileus, noncalcified, pneumobilia, rigler's triad

## Abstract

Gallstone ileus is a rare but serious complication of chronic cholecystitis, causing mechanical small bowel obstruction. Contrast-enhanced computed tomography (CT) plays a key role in radiological diagnosis. The classic findings are known as Rigler’s triad, comprised of pneumobilia, small bowel obstruction, and calcified gallstones. We report a unique case of a 74-year-old female patient who presented with hallmark clinical features of bowel obstruction. CT revealed bowel obstruction and pneumobilia but did not show calcified gallstones, deviating from the usual Rigler’s triad. Following midline laparotomy, a noncalcified gallstone was confirmed causing bowel obstruction. This case underscores the need to consider gallstone ileus in small bowel obstruction even in rare cases where conventional CT findings are not present, alongside the value of comprehensive radiological analysis and maintaining a high degree of clinical suspicion. Timely recognition of such atypical cases is vital for effective surgical treatment and better patient outcomes.

## Introduction

Gallstone ileus is a rare complication that occurs in approximately 0.5% of cases of chronic cholecystitis, resulting in mechanical small bowel obstruction. It occurs as a gallstone enters the small bowel through a biliary enteric fistula, with more than half of these being cholecystoduodenal fistulas. The ileum is the narrowest part of the bowel; therefore, the commonest site of stone impaction is at the ileocecal valve [1, 2]. While plain radiograph can be used for diagnosis, only a minority of gallstones have sufficient calcium content to be visible on abdominal X-rays; therefore, the gold standard for final diagnostic confirmation of gallstone ileus is with contrast-enhanced computed tomography (CT) with sensitivity and specificity of 90%-93% and 100%, respectively [3]. Typical CT findings are of Rigler’s triad: small bowel obstruction, gas within the biliary tree, and visualization of a gallstone typically in the right iliac fossa [4]. Here, we present a case of an elderly female patient presenting with hallmark clinical features of bowel obstruction; however, no calcified gallstones were seen on CT imaging as per Rigler’s triad. Only pneumobilia and small bowel obstruction were noted on the initial CT which raised suspicion of noncalcified gallstone ileus that was eventually confirmed surgically following diagnostic laparoscopy and then midline laparotomy.

## Case presentation

This case involves a 74-year-old female with a history of hypertension, type 2 diabetes, hypothyroidism, and diverticular disease, presenting to the emergency department with sudden-onset central abdominal pain 30 minutes postprandial. The pain was sharp and centralized, rated 7/10, and improved with recumbency but worsened with movement. The patient experienced dark brown vomiting, nausea, and no bowel movements since the onset of pain three days prior to admission. On examination, there was notable tenderness, guarding, and rebound tenderness in the right lower quadrant, along with absent bowel sounds. Lab data revealed leukocytosis alongside an elevated lactate and creatinine of 3.2 mmol/L and 145 mmol/L, respectively (Table 1).

**Table 1:** Admission blood tests including relevant point of care full blood count (FBC), venous blood gas (VBG), and liver function tests (LFTs): ALT and ALP ALT: Alanine transaminase; ALP: alkaline phosphatase

Test	Observed value	Reference range
FBCs		
Granulocytes	15.5 x 10^3^/µL	1.5-7.6 x 10^3^/µL
White blood cells	16.6 x 10^3^/µL	4-10 x 10^3^/µL
VBG		
Sodium	138 mmol/L	136-145 mmol/L
Potassium	3.4 mmol/L	3.4-5.1 mmol/L
Urea	12.5 mmol/L	2.1-7.1 mmol/
Creatinine	145 mmol/L	44-80 mmol/L
Lactate	3.2 mmol/L	0.5-2.2 mmol/L
pH	7.47	7.35-7.45
LFTs		
Bilirubin	40 mg/dL	0-20 mg/dL
ALP	137 U/L	30-130 U/L
ALT	25 U/L	0-55 U/L

**Figure 1:**
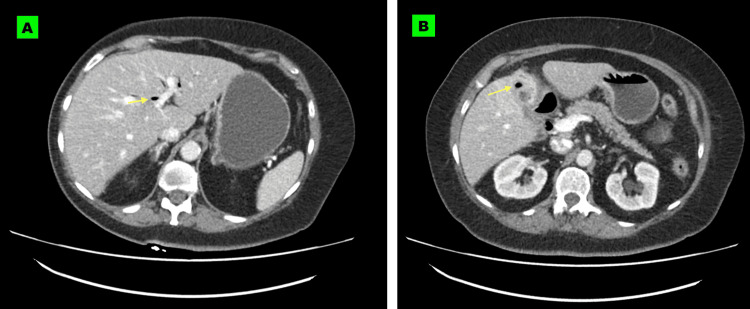
Axial CT slices of the upper abdomen show air in the biliary tree (pneumobilia) (A) and air in the gallbladder fossa (B) CT: Computed tomography

**Figure 2:**
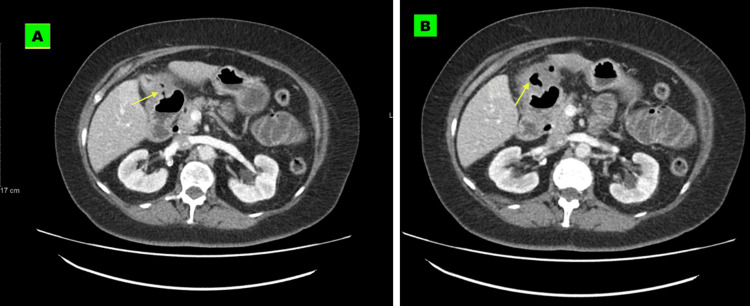
Axial CT slices at a lower level showing a track connecting the gallbladder with the duodenum consistent with cholecystodoudenal fistula (A and B) CT: Computed tomography

CT abdomen and pelvis with contrast was requested to rule out bowel obstruction and/or perforation, and while no evidence of calcification or increased density on CT was seen that would be suggestive of an obstructing gallstone, there was obvious pneumobilia and air in the gallbladder fossa (Figures 1A-1B) and an air-filled track connecting the gallbladder with the proximal duodenum suggestive of a cholecystoduodenal fistula (Figures 2A-2B).

**Figure 3:**
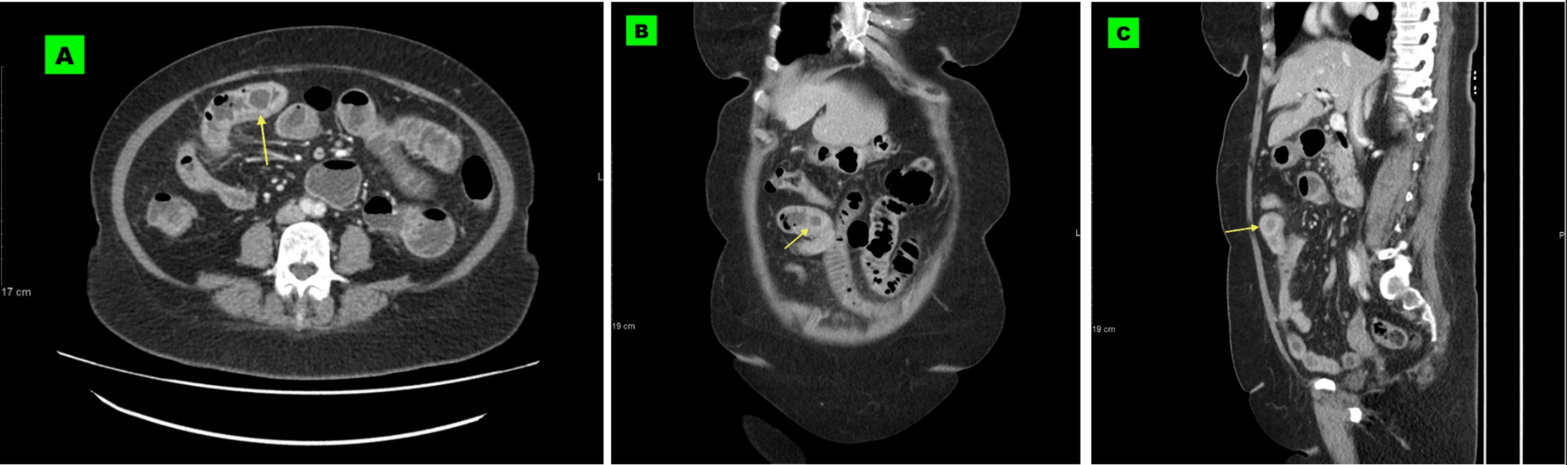
Axial (A), coronal (B), and sagittal (C) CT images show dilated small bowel loops with a well-defined isodense lesion at the transition point (arrows), which shows faint calcification best seen on the sagittal reconstruction. This was surgically proven to be a large gallstone CT: Computed tomography

These findings, coupled with obvious dilatation of the jejunum and proximal ileum (Figures 3A-3C) ruled in the potential diagnosis of gallstone ileus. 

**Figure 4:**
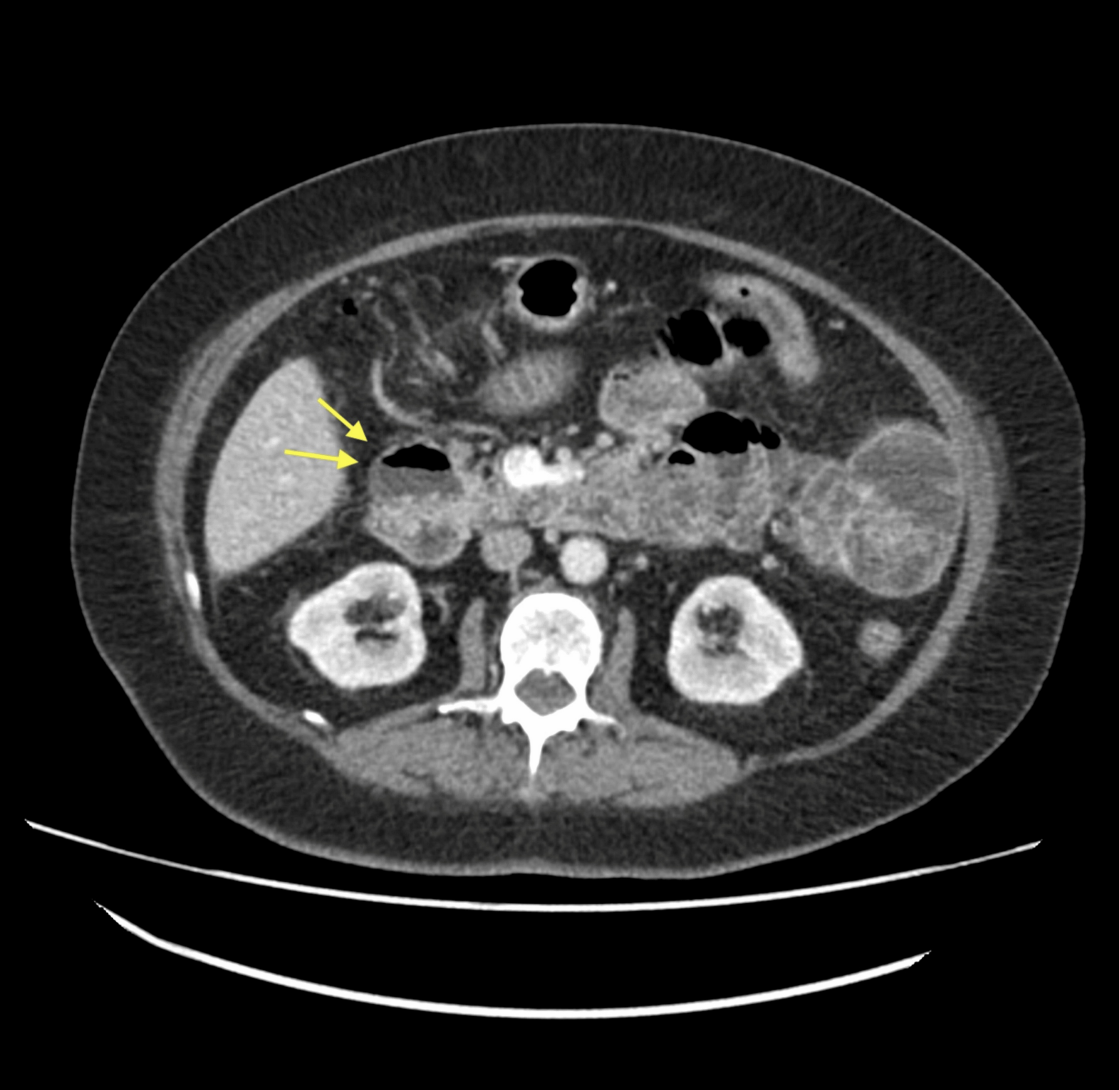
Axial CT slice shows an incidental finding of a duodenal diverticulum, arising from the second part of the duodenum CT: Computed tomography

To note, an incidental finding of a duodenal diverticulum was found (Figure 4).

This patient underwent midline laparotomy following the CT, and a noncalcified gallstone was found impacted at the proximal ileum causing upstream dilatation of the jejunum with three areas of sealed perforations in the ileum. The surgically proven gallstone was 3 cm in diameter. 

## Discussion

Gallstone ileus is a serious cause of mechanical bowel obstruction. A typical presentation is colicky abdominal pain, either generalized or localized to the right upper quadrant, followed by nausea and vomiting [3]. Symptoms vary in duration from hours to weeks and may include less common features like constipation and abdominal distension [5]. As mentioned previously, the investigation of choice is contrast-enhanced CT looking for the classic features that make up Rigler’s triad. In often cases, the triad will not be seen especially on other imaging modalities such as plain radiograph; however, what is more consistently seen is some form of opacification on CT due to partial or complete calcification of the impacting gallstone even if other aspects of Rigler’s triad may not be present. Literature suggests dense opacification is seen in 48.3% of cases, slight opacification in 11.5%, rim opacification in 21.8%, and radiolucent or pure cholesterol stones in 14.9% [6]. The identification of gallstones on CT is complicated by variability in composition and structure. In such difficult cases, the diagnosis depends on a high index of suspicion and the skill of the observer. 

In this case, we found only two features of Rigler’s triad present on CT: evidence of small bowel obstruction highlighted by dilatation of the jejunal and ileal loops up to 4 cm in diameter alongside pneumobilia. Although pneumobilia is a sign highly suggestive of gallstone ileus, its presence could also be due to a previous sphincterotomy or post-endoscopic retrograde cholangiopancreatography (ERCP). Other differentials to consider in this case were adhesions or intussusception. Adhesions or adhesive bands are the most common cause of mechanical bowel obstruction in the developed world, and the diagnosis could be challenging in this case due to an iso-attenuating stone relative to the fluid, which has accumulated due to the obstructed bowel [7,8]. This highlights a notable drawback of intravenous contrast-enhanced CT scan as it can be difficult to define some radiolucent stones or rim calcified stones which may resemble soft tissue densities such as a mass or intussusception.

The radiological diagnosis of gallstone ileus certainly changed the management pathway in this case, and played a key role in early appropriate escalation for prompt surgical intervention. This case report underscores the importance of recognizing atypical presentations of gallstone ileus on CT imaging, particularly when calcified stones are not visualized. Clinicians should maintain a high index of suspicion, relying on clinical correlation in cases where imaging does not align with the classic Rigler's triad. The report emphasizes the need to consider uncommon causes of small bowel obstruction and highlights the significance of thorough evaluation beyond typical scenarios. The management of gallstone ileus is predominantly surgical with removal of the obstructing stone via enterolithotomy, usually followed by a cholecystectomy or fistula closure to treat the cause [9]. Bowel resection is not uncommon if surgical complications occur such as perforation or difficult stone retrieval [10,11]. On the other hand, adhesions, the most common cause of mechanical bowel obstruction, are often managed conservatively to prevent new adhesions [7,8]. Therefore, it is crucial to differentiate and consider the diagnosis of gallstone ileus, even in the absence of calcification on imaging, as this would significantly alter the management approach.

## Conclusions

The prompt diagnosis and treatment of gallstone ileus are key in optimizing outcomes for patients, especially elderly patients with multiple comorbidities who are at high risk of mortality and significant morbidity. While radiolucent calcification of gallstones is usually visible on CT, the absence of calcified stones can occur and necessitates a high index of suspicion and thorough clinical correlation, especially in the context of other radiological features such as pneumobilia and small bowel obstruction. Surgical intervention remains the definitive treatment, emphasizing the need for prompt and effective management to prevent complications. This report aims to enhance awareness among clinicians and radiologists about the variability in presentations of gallstone ileus, thereby improving diagnostic accuracy and patient outcomes.
